# Recognition of Crop Diseases Based on Depthwise Separable Convolution in Edge Computing

**DOI:** 10.3390/s20154091

**Published:** 2020-07-22

**Authors:** Musong Gu, Kuan-Ching Li, Zhongwen Li, Qiyi Han, Wenjie Fan

**Affiliations:** 1 College of Information Science and Technology, Chengdu University, Chengdu 610106, China; msgu@cdu.edu.cn (M.G.); hanqiyi@cdu.edu.cn (Q.H.); fanwenjie@cdu.edu.cn (W.F.); 2 School of Information and Communication Engineering, University of Electronic Science and Technology of China, Chengdu 611731, China; 3 Key Laboratory of Pattern Recognition and Intelligent Information Processing, Institutions of Higher Education of Sichuan Province, Chengdu University, Chengdu 610106, China; 4 Department of Computer Science and Information Engineering, Providence University, Taichung 43301, Taiwan; 5 School of Computer Science and Engineering, Anhui University of Science and Technology, Huainan 232001, China

**Keywords:** depthwise separable convolution neural network, recognition of crop diseases, Visual Geometry Group (VGG) network model

## Abstract

The original pattern recognition and classification of crop diseases needs to collect a large amount of data in the field and send them next to a computer server through the network for recognition and classification. This method usually takes a long time, is expensive, and is difficult to carry out for timely monitoring of crop diseases, causing delays to diagnosis and treatment. With the emergence of edge computing, one can attempt to deploy the pattern recognition algorithm to the farmland environment and monitor the growth of crops promptly. However, due to the limited resources of the edge device, the original deep recognition model is challenging to apply. Due to this, in this article, a recognition model based on a depthwise separable convolutional neural network (DSCNN) is proposed, which operation particularities include a significant reduction in the number of parameters and the amount of computation, making the proposed design well suited for the edge. To show its effectiveness, simulation results are compared with the main convolution neural network (CNN) models LeNet and Visual Geometry Group Network (VGGNet) and show that, based on high recognition accuracy, the recognition time of the proposed model is reduced by 80.9% and 94.4%, respectively. Given its fast recognition speed and high recognition accuracy, the model is suitable for the real-time monitoring and recognition of crop diseases by provisioning remote embedded equipment and deploying the proposed model using edge computing.

## 1. Introduction

With the development of Internet of Things (IoT) technology, systems based on the IoT are often used in environmental monitoring because of its low cost and secure deployments, such as forest fire monitoring, crop growth monitoring, and marine climate monitoring [1]. In the process of system deployment, sensors are added to the system, followed by large-scale data processing analysis and storage that brings many challenges to the IoT system based on a cloud platform, such as real-time feedback, bandwidth load, and network connection stability, among others. With advances in communication and processing technologies, edge computing technology is rapidly incorporated into IoT monitoring systems [2], where edge computing node devices as the intelligent gateway, lightweight server, and small base station are physically located in the middle layer of the system, closer to the IoT terminal devices than the cloud platform. Therefore, edge computing nodes can provide local services to terminal devices to improve the real-time performance of services and information feedback, and thus reduce the return time delay caused by the remote interaction between terminal devices and cloud platform. Concurrently, edge computing devices can also provide preliminary data processing, divert computing tasks from the cloud platform, reduce the amount of data uploaded to the cloud platform, and reduce the bandwidth load of the backbone link [3,4,5,6,7,8].

At present, crop disease detection methods mainly include spectroscopy and imaging, hyperspectral imaging, and other technologies. The diagnosis and identification of crop disease can be realized by establishing an infection model of crop disease. However, it is not suitable for large-scale crop disease monitoring due to the need for manual collection of experimental samples, expensive experimental equipment, and consumables and other reasons. At the same time, with the development of technologies for agriculture, image recognition is fashionable to detect crop diseases. The convolution neural network (CNN) is often used in image detection because of its strong learning ability and has higher accuracy for image classification of vegetable diseases.

In Reference [9], various crop diseases are classified by a CNN with high accuracy. A detection method of cotton disease images based on CNN is proposed in Reference [10] and achieves better results. During the detection of vegetable diseases, CNN is prone to overfitting due to its complex structure parameters. The transfer learning based on the Visual Geometry Group (VGG) network model is proposed in Reference [11] to improve the image classification accuracy of crop diseases and ease the overfitting phenomenon, where the image features of tomato diseases are extracted through the VGG network model and classified through support vector machine (SVM) to detect tomato diseases, and excellent results are achieved. However, current research can only recognize and detect crop diseases as soon as an emergency of the uncontrolled situation is installed, which dramatically delays the treatment period of crop diseases and causes enormous economic losses. Therefore, there is a need to identify and detect crop diseases in a timely and accurate manner when they are emerging.

In this article, we present the IoT monitoring system framework based on edge computing and discuss the functions of components and the communication process between them in detail. As the system framework, we propose a method of detecting crop diseases using a depthwise separable convolutional neural network (DSCNN), in which lightweight characteristics make it highly suitable for deployment in edge devices. DSCNN can process complex data models and not only model and analyze linearly dependent data, but also process non-linearly dependent data. Aimed at the complexity of neural network model training, the training process is deployed on the cloud platform, and the obtained model and parameters will be sent back to the edge computing nodes for subsequent disease detection. At the edge computing nodes, we use the camera to take regular and random pictures of the crops, recognize the images, and give an alarm on time if diseases and insect pests are discovered. Experimental results show that the detection method of crop diseases based on the proposed DSCNN model can accurately detect crop diseases and avoid substantial economic losses.

This article is organized as follows. Section 2 introduces the monitoring system based on edge computing, while the proposed DSCNN model is described in Section 3. In the Section 4, computational results and comparison to other convolutional neural networks are presented, and finally, concluding remarks and directions for future work are depicted in Section 5.

## 2. Monitoring System Based on Edge Computing

**Figure 1:**
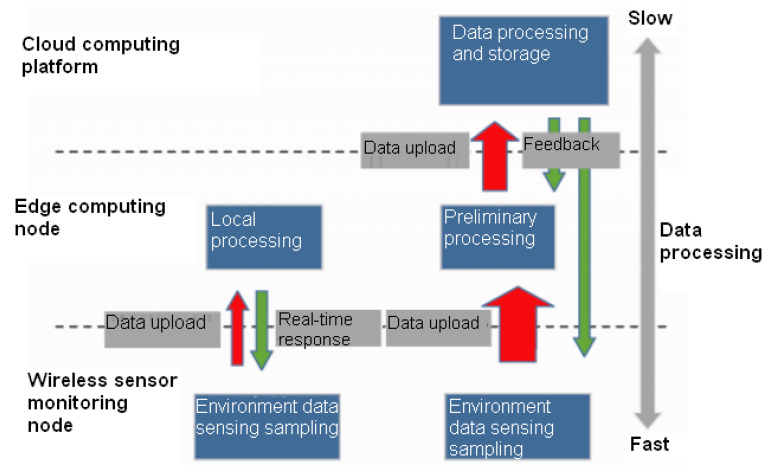
The monitoring system for crop diseases and insect pests.

As shown in Figure 1, the monitoring system based on Edge computing is composed of edge computing nodes, sensor nodes, and a cloud computing platform.

### 2.1. Sensor Node

The wireless sensor node is the most basic and essential part of the traditional IoT monitoring system [12]. A large number of nodes are randomly or systematically arranged in the monitoring area to sense and collect environmental data in real-time. For example, in the forest monitoring system GreenOrbs deployed in Wuxi, Jiangsu Province (China), wireless sensor nodes are placed on trees, and each node is embedded with temperature, humidity, light intensity, and carbon dioxide concentration sensors to monitor the forest environment and detect and prevent forest fire in real-time [13]. In the IoT system framework based on edge computing, wireless sensor nodes are mainly used to monitor the growth of crops and to communicate with edge computing nodes. On the one hand, it can reduce the requirement for wireless sensor nodes; on the other hand, it can make full use of the data processing ability of edge computing nodes [14].

### 2.2. Edge Computing Node

In the IoT monitoring system, small ground base stations deployed around the monitoring nodes can be taken as edge computing nodes. The edge computing node plays an essential role in the proposed recognition and detection method of diseases and insect pests. The deep neural network model trained by the cloud computing center is deployed to the edge computing node in the system’s initial stage. The collected data are recognized and detected in the normal operation stage of the system. Once an exception is detected, the edge computing node will immediately report the exception to the data and control center located on the cloud platform and drive the controller at the bottom to provide an emergency response plan.

### 2.3. Cloud Computing Platform

Because the initial model training of deep neural network requires a massive amount of data and computation, which is difficult for the edge computing node to bear, we conducted the model pre-training and learning on the cloud computing platform [15] and deployed the generated model parameters to the edge computing node. Based on these model parameters, edge computing nodes can run the proposed algorithm that will significantly reduce the time of pre-training and greatly improve the prediction accuracy.

## 3. Proposed Algorithm

### 3.1. Recognition of Crop Diseases

Using CNN technology to apply “prior knowledge” to the learning process supports solving the small samples of crop diseases [16]. The basic principle is as follows: with the transfer learning (TL), the “prior knowledge” obtained from “non-single” datasets is applied to CNN training of domain-specific recognition to alleviate the overfitting problem caused by insufficient data volume in a specific domain. In this paper, TL classifies crop diseases to apply the useful skills learned from one or more auxiliary domain tasks to the new targets and tasks [17,18,19,20,21,22,23]. Most of the research where the TL method is applied to the recognition of crop diseases is based on the TL method for parameter fine-tuning. The combination of a depthwise separable convolutional network and transfer learning for the recognition of crop diseases can improve recognition accuracy and improve disease recognition efficiency. It can also be applied to intelligent terminal devices in a better way.

### 3.2. CNN

**Figure 2:**
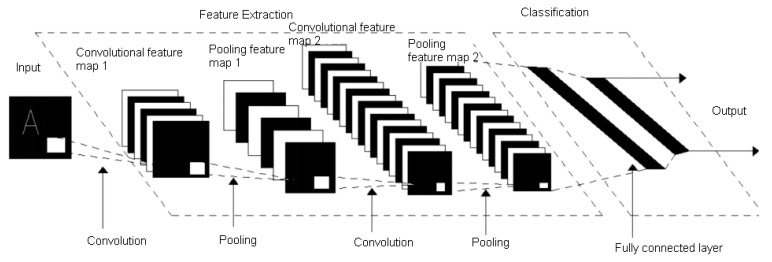
Convolution neural network (CNN) structure diagram.

In the deep learning model, CNN is mainly used for image and speech recognition. It is a weight-sharing neural network structure and consists of an input layer, convolutional layer, pooling layer, fully connected layer, and output layer [24,25,26], as shown in Figure 2.

(1) Convolutional layer

Also known as convolution kernels, the core component of CNN consists of a set of learnable filters, where each convolution kernel convolves the inputs. For example, a two-dimensional image *I* is taken as the input and the convolution kernel function is expressed as *K*, and the image *I* and convolution kernel *K* are convolved as:

(1) $$S(i,j)=(I*K)(i,j)=\sum_{m}\sum_{n}I(m,n)K(i-m,j-n)$$

where *S(i,j)* is the matrix of the image *I* and convolution kernel *K* after convolution operation (the convolution operator is denoted by “*”), *m* and *n* denote the number of pixel points on the image *I*, and *i* and *j* denote the matrix size after convolution operation, respectively. The matrix is a convolution feature of the original image. 

The image is convolved with the convolution kernel and then undergoes nonlinear transformation to get the feature map, expressed as:

(2) $$a^{i,l}=f(z^{i,l})=f(W^{l}*a^{i,l-1}+b^{i,l})$$

where *z^i,l^* denotes the weighted sum of the *i*th sample on the *l*th layer, *a^i,j^* denotes the output of the *i*th sample on the *l*th layer, * denotes the convolution operation, *W^l^* denotes the weight of the convolution kernel on the *l*th layer, *b^i,l^* denotes the bias of the *i*th sample on the *l*th layer, and *f*(•) denotes the activation function of neurons. Rectified Linear Unit (ReLU) is often used in the convolutional neural network and expressed as:

(3) f(x)=max(0,x)

It is noted that the process of obtaining the feature map *a^l^* of the current layer is as follows: perform the convolution operation between *W^l^* and feature map *a^l−1^* of the l-1st layer in CNN, and then add the result to the bias vector *b^l^* of the current layer, and finally complete the nonlinear transformation by the nonlinear excitation function.

(2) Pooling layer

The main principle is as follows: different parts of the image can be aggregated, and the output of the network at a particular location can be replaced by the overall statistical features of the adjacent output at that location. The pooling process is expressed mathematically as:

(4) $$a^{i,l}=pool(a^{i,l-1})$$

(3) Fully connected layer

The input images are alternately extracted and dimensioned through multiple convolutional layers and pooling layers. Moreover, they are connected with the fully connected network to classify the features and realize the mapping of the probability distribution from the input image to the category, and are mathematically expressed as:

(5) $$a^{i,L}=soft\max(z^{i,L})=\frac{e^{W^{L}•a^{i,L-1}+b^{i,L}}}{\sum {}_{j}e^{W^{L}•a^{j,L}+b^{j,L}}}$$

where *L* denotes the output layer, softmax function is equivalent to a classifier, and the output image corresponds to the probability of the category.

CNN mainly includes two stages: forward propagation and back propagation.

i. Error back propagation of the output layer

We adopt the standard mean square deviation function to measure the loss, expressed mathematically as:

(6) $$J(W,b)=\frac{1}{2}||a^{i,L}-y|{|}_{2}^{2}$$

where *a^i,L^* denotes the output of the *i*th sample on the output layer, *y* denotes the sample label, and ||•|| is the L2 norm of •.

ii. Error back propagation of the pooling layer

*δ^i,l^* denotes the gradient error of the *i*th sample on the *l*th layer. If *δ^i,l^* of the pooling layer has been known, we can derive *δ^i,l−1^* of the previous convolutional layer. The error term of a sample is expressed as:

(7) $$\delta^{i,l-1}=upsample(\delta^{i,l})⊙f^{\prime }(z^{i,l-1})$$

where, *upsample()* is the upsample function, and “0” denotes the dot product of a matrix.

iii. Error back propagation of convolutional layer

If *δ^i,l^* of the convolutional layer has been known, we can derive *δ^i,l−1^* of the previously hidden layer and express it as:

(8) $$\delta^{i,l-1}=\delta^{i,l}*rot180(W^{l})⊙f^{\prime }(z^{i,l-1})$$

where “rot180” denotes 180° rotation of the convolution kernel.

iv. Calculation of weight and bias gradient using error term

By solving the error terms of the output layer, convolutional layer, and pooling layer by error back propagation, we can calculate the gradient of the loss function for parameters in CNN according to error terms. Since the pooling operation is only performed on the pooling layer and there are no parameters involved in the operation, we only need to calculate the gradients of the fully connected and convolutional layers. The gradient calculation of the fully connected layer is actually to solve the derivatives of the weight and the bias between feature vector and output vector respectively, expressed as:

(9) $$\frac{\partial J(W,b)}{\partial W^{L}}=[(a^{i,L}-y)⊙f^{\prime }(z^{i,L})]{\left(a^{i,L-1}\right)}^{T}$$

(10) $$\frac{\partial J(W,b)}{\partial b^{L}}=(a^{i,L}-y)⊙f^{\prime }(z^{i,L})$$

where *T* in the upper right corner denotes the transposition operation. 

To sum up, the training method of CNN is based on error back propagation and gradient descent algorithms. The difference is just the way of derivation between layers. By calculating each layer’s corresponding error term, we can retrieve the parametric derivative of each layer and, finally, adjust each layer’s parameters using the gradient descent algorithm.

### 3.3. VGG Network Based on Transfer Learning

**Figure 3:**
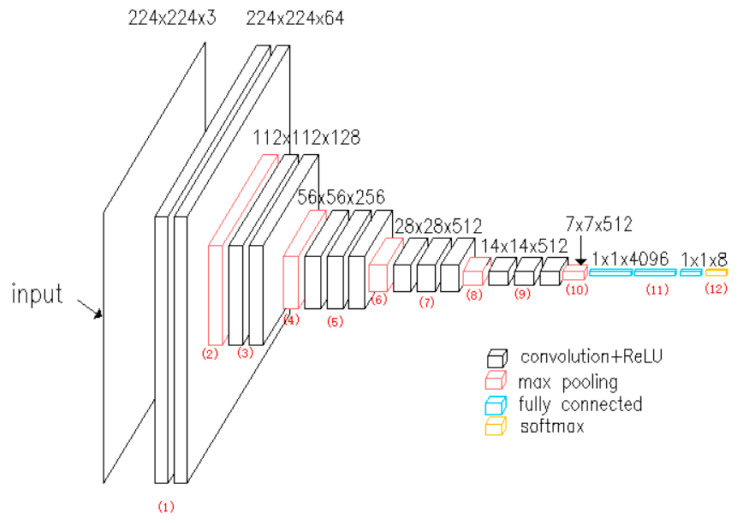
Diagram of VGG network.

According to the types and features of tomato diseases, a recognition model for eight tomato diseases is constructed based on the VGG network. The VGG network is shown in Figure 3. The trained VGG network can be used as the pre-training model to reduce network training time and improve network training efficiency. The number of weight parameters is 65 × 103 and the parameters are designed for 1000 classification categories. In this paper, according to the study’s actual situation, the classification categories are changed to 8, corresponding to 8 tomato diseases and insect pests.

### 3.4. Recognition Model Based on Depthwise Separable Convolutional Network

To optimize the limited computing resources of edge node devices, the VGG network based on standard convolution is improved by a depthwise separable convolutional (DSC) network to reduce the parameters and calculation amount of model feature extractor.

**Figure 4:**
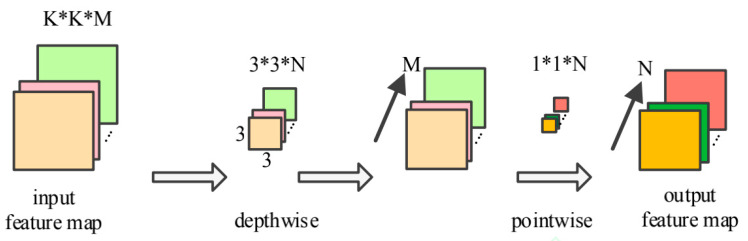
Depthwise separable convolution (DSC).

DSC is the main structure of the lightweight neural network, and its primary function is to reduce the network parameters, compress the network structure, reduce the calculation amount, and improve the operation speed of the network while ensuring the network nonlinearity and making full use of the feature information [27,28,29]. The traditional convolution process is that the input image is convolved with the convolution kernel of the same depth to get the feature information. The DSC is composed of depthwise and pointwise, whose structure is shown in Figure 4.

The process of depthwise separable convolution can be divided into two parts: Depthwise convolution (Conv dw) and Pointwise convolution (Conv pw). Depthwise refers to the convolution operation of a 3 × 3 single-channel convolution kernel on each channel of the corresponding input data. Pointwise refers to the convolution operation of a 1 × 1 convolution kernel (the number is the number of output channels). Among them, M and N denote the number of input channels and the number of output channels. The DSC has the same output feature dimension as the traditional convolution, though it dramatically reduces the model’s parameters and calculation amount. 

Convolution operation occupies the vast majority of computation in the neural network, while matrix multiplication in convolution operation occupies most of the computation in convolution operation. Considering that the input size of a convolutional layer is *C_i_* × *H_i_* × *W_i_*, the convolution kernel is *C_o_* × *K_H_* × *K_W_*. The output feature map is *C_o_* × *H_o_* × *W_o_* (where *C* denotes the number of channels, *H* and *W* denote the height and width, and *i* and *o* denote the input and output), the calculation process of activation layer and bias is ignored, and the calculation amount of conventional convolution is estimated as:

(11) $$F_{Conv}=C_{i}\times C_{o}\times H_{o}\times W_{o}\times K_{H}\times K_{W}$$

The calculation amount of DSC is:

(12) $$F_{DS-Conv}=C_{i}\times K_{H}\times K_{W}\times H_{o}\times W_{o}+C_{i}\times C_{o}\times H_{o}\times W_{o}$$

The ratio of the two is calculated as:

(13) $$\frac{F_{DS-Conv}}{F_{Conv}}=\frac{C_{i}\times K_{H}\times K_{W}+C_{i}\times C_{o}}{C_{i}\times C_{o}\times K_{H}\times K_{W}}=\frac{1}{C_{o}}+\frac{1}{K_{H}+K_{W}}$$

**Figure 5:**
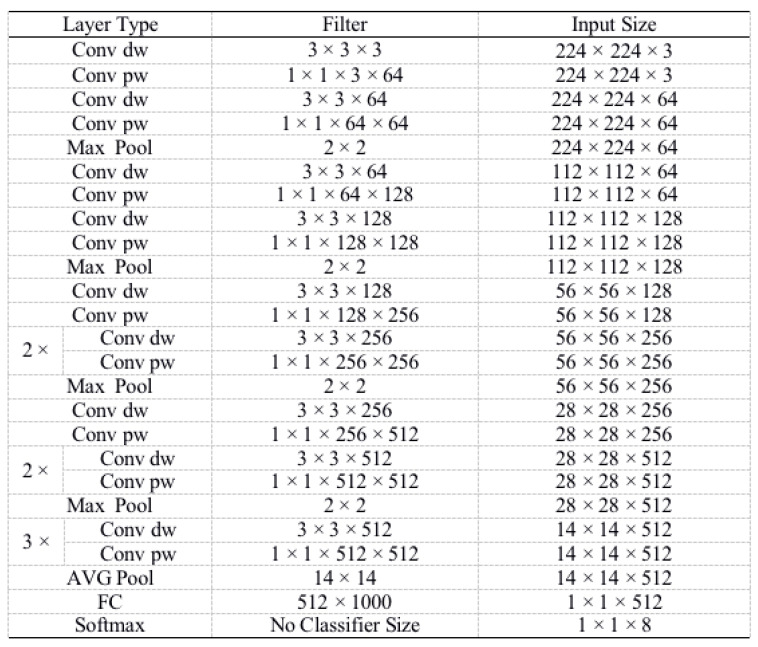
Depthwise separable convolutional neural network (DSCNN).

As can be seen from Equation (13), the DSC can reduce the calculation amount. DSC improves the VGG network based on standard convolution. It can reduce the size of the model and achieve the same convolution effect as the standard convolution so that it can be used for image classification, detection, segmentation, and other tasks as the large-scale feature extraction model. The improved core network is a depthwise separable convolutional neural network composed of a separable convolutional layer and Max Pool layer (average pooling layer). The size of an input image is 224 × 224. After a series of separable convolutions and the processing of Max Pool, full connection (FC) and Softmax classifier, 8-dimensional features are finally output as the calculation and analysis results of category 8, as shown in Figure 5.

### 3.5. Algorithm Flow Chart

**Figure 6:**
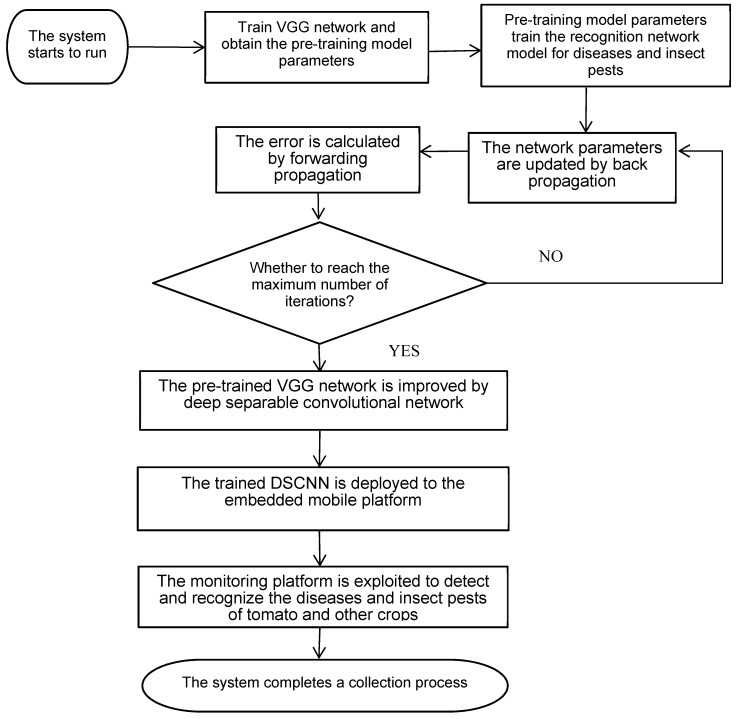
Crop diseases and insect pests detection system flow chart.

The flow chart for a crop diseases and insect pests detection system is shown in Figure 6.

Step1: Based on transfer learning, the cloud computing platform uses the ImageNet database to train the VGG network and obtains the pre-training model parameters.

Step2: Pre-training model parameters train the recognition network model for diseases and insect pests, and the error is calculated by forward propagation and the network parameters are updated by back propagation. The program performs the preset number of iterations, and such model’s operation is terminated as soon as the maximum number of iterations is reached.

Step3: The pre-trained VGG network is improved by the depthwise separable convolutional network to reduce the parameters and calculation amount of the model feature extractor.

Step4: The trained depthwise separable convolutional network is deployed to the embedded mobile platform.

Step5: The monitoring platform is exploited to detect and recognize the diseases of tomato and other crops. If the related crop diseases are recognized, relevant data and information shall be sent back to the background server via Sink node to promptly grasp the growth of crops and the situation of crop diseases.

Step6: Once the system completes a collection process, the node enters the monitoring state again.

## 4. Results and Discussion

### 4.1. Experimental Environment

Experiments were performed on a server system composed of one Intel i7 Core 2.20 GHz CPU, 16 GB memory and one NVIDIA GTX1060 card, with Win10 operating system and Python 3.6 installed. The DSCNN was used as the feature extraction network of crop diseases, and the Softmax multi-category classifier was used for classification. The advantages and disadvantages of the proposed algorithm were analyzed compared with the traditional convolutional neural network in terms of recognition accuracy and detection speed.

**Figure 7:**
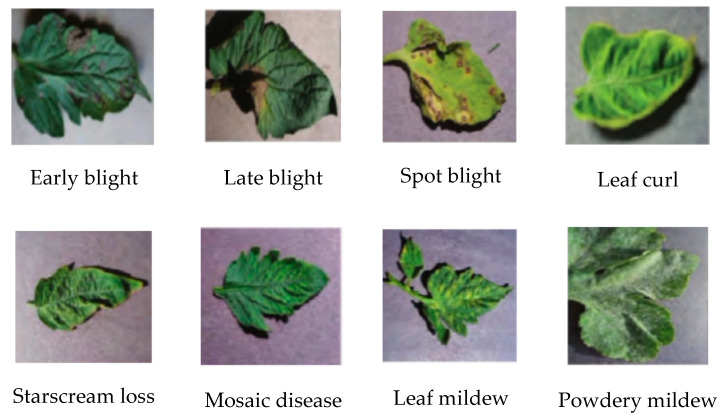
The eight tomato diseases.

The tomato diseases’ image samples selected in this paper are from Plant Village, which contains an extensive dataset of professional agricultural pictures. The leaf images of eight common tomato diseases were selected, and 600 pictures were selected for each category, totaling 4800 pictures. In each category of images, 600 images were divided into two parts, among which 480 images belong to the training set and 120 images belong to the test and verification set. The resolution of the collected images was about 224 × 224 × 3 pixels. The eight tomato diseases are as follows: early blight, late blight, spot blight, yellow leaf curl disease, starscream loss, mosaic disease, leaf mildew, and powdery mildew, as shown in Figure 7. The above leaf diseases are mainly formed by fungal infection of Streptococcus solanopsis, Phytophthora infestans, and other fungi. The main symptoms are producing disease spots or mildew on the leaves or stems.

The leaf images were pre-processed to produce better detection results. Due to the different sizes of the pictures that contain much redundant information, image samples of tomato disease and insect pest leaves were normalized to 224 × 224 × 3 pixels.

### 4.2. Visual Feature Map

**Figure 8:**
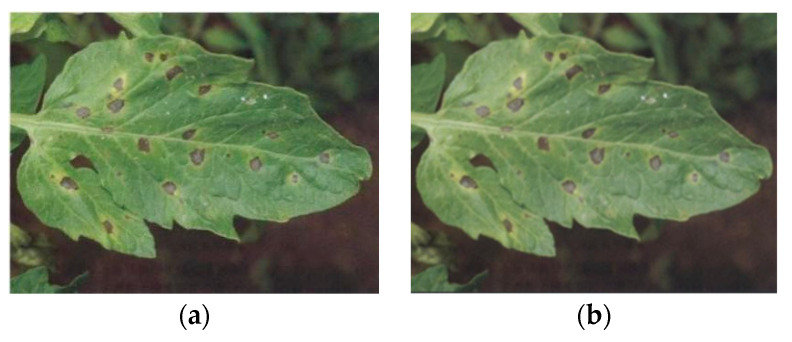
Original image and pre-processed image of tomato leaf mildew. (**a**)The Original image of tomato leaf mildew, (**b**) The pre-processed image of tomato leaf mildew.

The convolutional layer aims to process the feature extraction of pictures. In this section, we introduce how to extract the features of tomato diseases by convolutional layers in a visual way. Each convolutional layer will extract the features of tomato disease and insect pest leaves. To see the effect of model extraction more intuitively, we took an image of tomato leaf mildew as an example to train the model and visualize the convolution kernel output of each layer. Figure 8 is the original image and the pre-processed image of tomato leaf mildew.

**Figure 9:**
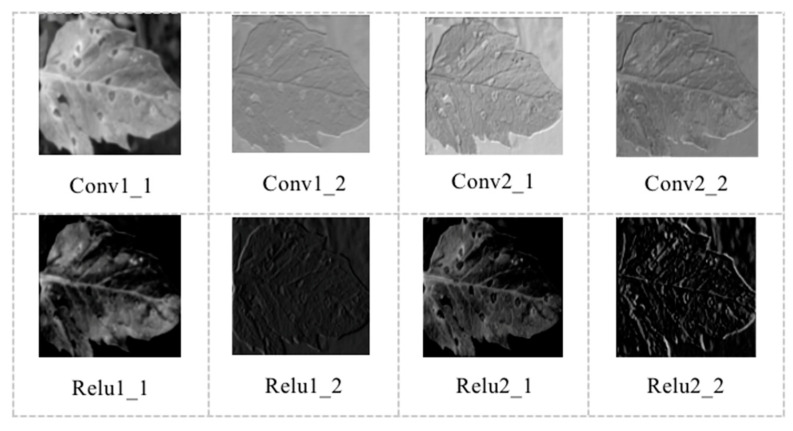
Output features of Conv1-2 and Relu1-2 layers of tomato diseases.

Figure 9 shows the output features of convolutional layers 1-2 (Conv1-2) and relu layers 1-2 (Relu1-2) of tomato diseases. As shown in Figure 9, the contour features of tomato disease and insect pest leaf images can be displayed by different color channels. Most of the convolutional kernel content is the contour edge information of the leaf image, and the convolutional kernel retains all image information.

**Figure 10:**
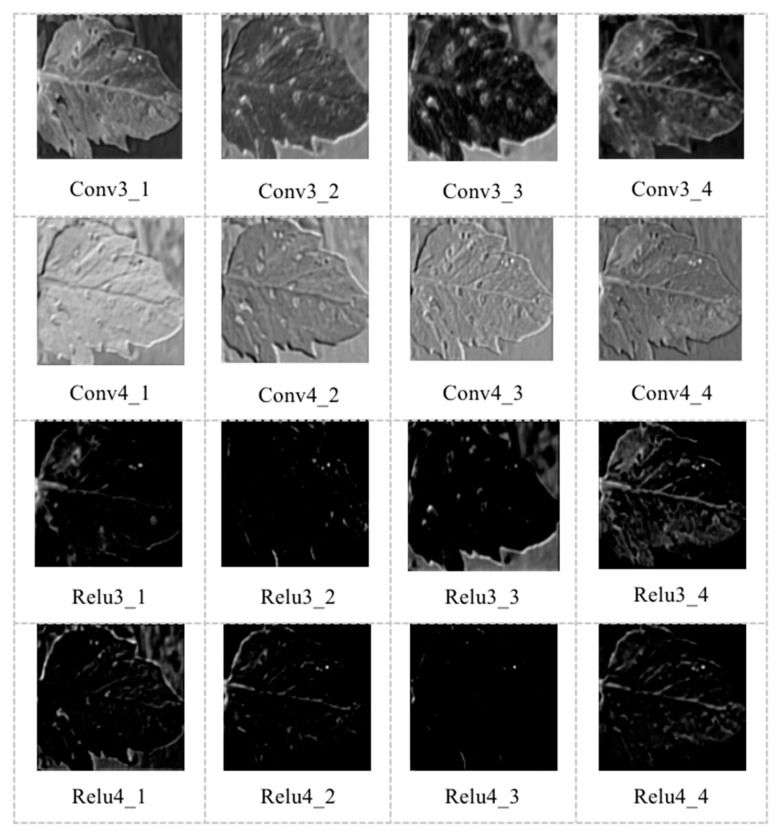
Output features of Conv3-4 and Relu3-4 layers.

Figure 10 shows the output features of convolutional layers 3-4 (Conv3-4)and relu layers 3-4 (Relu3-4) layers. In the output feature image, the contour of the image is more visible, while the image’s resolution is increasingly smaller. With the increase in the number of layers, the convolutional kernel’s output content is more abstract, and the retained information is gradually less.

**Figure 11:**
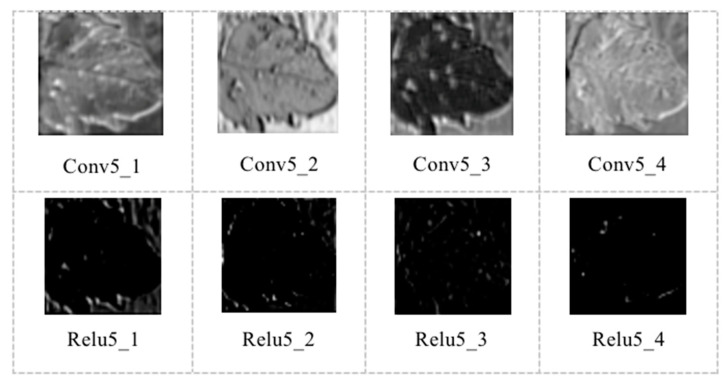
Output features of Conv5 and Relu5 layers.

Figure 11 shows the output features of conv5 and relu5 layers. The output feature map of the network has been blurred, with more blank content. However, careful observation shows that some areas in the output feature map are more straightforward to distinguish than other areas of the image, since the deep network can extract the most stable features of the image by integrating the underlying features.

As seen from the above feature map, the underlying network extracts the contour, shape, and surface information of the leaf. With the deepening on the number of layers, the features extracted from the network are more representative, to extract the most stable features of the leaf.

### 4.3. Analysis and Comparison of Experimental Results

#### 4.3.1. The Recognition Accuracy of the DSCNN Model

To fit the training network better, the learning rate was 0.01, the number of iterations was 400, the number of training images input in batches was 100, and the size of the pre-input image was 224 × 224 when training the network model. The abscissa Epoch unit in the figure refers to complete learning of the training set.

**Figure 12:**
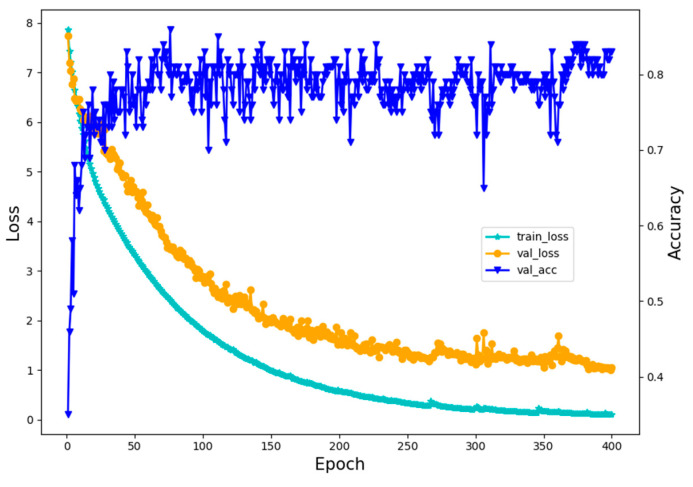
Prediction accuracy and loss function value of the DSCNN model.

Figure 12 illustrates the prediction accuracy and loss function value of the DSCNN model changing with the number of iterations. Experiments were carried out for 400 iterations, in which the blue line represents the prediction accuracy of the model under the validation set, the green line represents the loss function value of the model under the training set, and the orange line represents the loss function value of the model under the validation set.

The corresponding prediction accuracy and loss function values are output in the train and validation stages, respectively. As depicted in Figure 12, the prediction accuracy only occurs in the validation stage of the network model, while the loss function value occurs in both the training and validation stage. In terms of prediction accuracy, the DSCNN model has reached a high accuracy at the start of the test. During the first 20 iterations, the model’s testing accuracy is 0–70%, improving the prediction accuracy of the model rapidly, next exceeding 80% and reaching up to 90% by the end of the experimentation.

In terms of loss function value, the model’s loss function fluctuates significantly in the training stage, even though the overall trend is to decrease with the increase of the training period. The loss function value was relatively stable in the testing stage, but the overall trend was to decrease with the increase of the testing period. According to the above experimental results, the model can achieve higher prediction accuracy and speed in a short time after training and can meet the image recognition requirements of embedded devices for edge computing.

#### 4.3.2. The Recognition Accuracy of Various Crop Diseases

**Figure 13:**
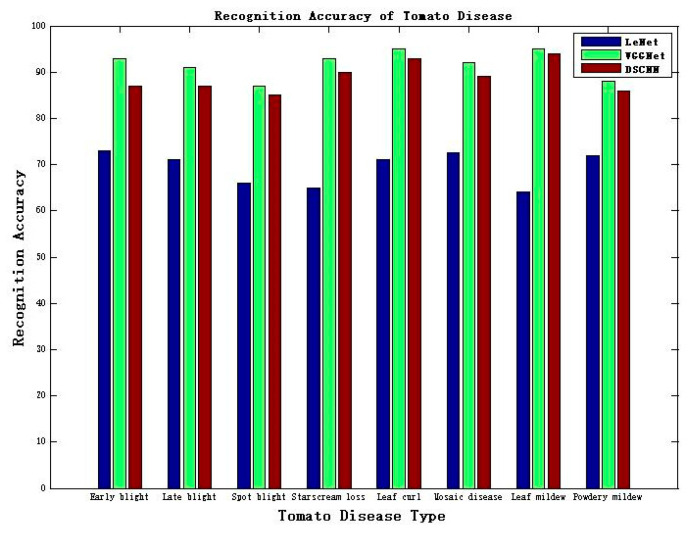
Comparison of accuracy of tomato diseases between LeNet, VGGNet, and DSCNN.

In order to evaluate the performance of the proposed model further, the classical convolutional neural network models LeNet and VGGNet were compared with the proposed DSCNN. Of the images collected in the image database, 80% were selected as training samples, and 20% as test samples. The accuracy results of the training samples and test samples are shown in Figure 13. To compare and analyze the advantages and disadvantages of the proposed DSCNN model with other deep learning neural networks, we conducted the experiments based on the existing datasets under the validation dataset.

The VGG model’s accuracy exceeds 91% in training sample recognition because the VGGNet network model has excellent generalization ability, which can accurately extract the necessary features. The average recognition accuracy of the DSCNN model is more than 89%, and the recognition accuracy is slightly worse than that of the VGGNet network. However, the recognition accuracy of the DSCNN model is much higher than that of the traditional deep learning neural network LeNet. Experimental results show that the average recognition accuracy of LeNet is about 69%, and the recognition accuracy of the DSCNN model is about 20% higher than that. 

#### 4.3.3. Comparison of Recognition Speed

To compare the overall performance of various network models under the dataset, we compared the separable convolutional neural network with the traditional deep neural networks, LeNet and VGG, including the average recognition accuracy and algorithm prediction speed under the same data. The specific experimental results are shown in Table 1.

The depthwise separable convolution neural network dramatically simplifies the model parameters due to its network structure features and improves the speed of identifying disease and insect pest images in the model recognition stage. Depthwise and pointwise operations are added compared with the traditional convolution operation, which significantly reduces the model’s parameters and calculation amount. Therefore, compared with the VGGNET and LeNet models, the prediction speed of DSCNN was the fastest, which was 0.239 s.

At the same time, due to transfer learning in the training and test stages, the DSCNN model shows a proper state distribution in the terminal output, weight parameters, and bias parameters, and the errors generated in the shallow network parameter training will not be infinitely magnified in the deep layer, so the model is increasingly stable.

Overall, experiments on high recognition accuracy show that the method proposed in this research significantly improves the recognition speed. Compared with LeNet and VGG models, the recognition time is reduced by 80.9% and 94.4%, respectively. Based on the significant increase in recognition speed, the DSCNN model is more suitable for deploying resource-limited edge computing equipment, which dramatically improves the real-time performance of pest and disease recognition.

## 5. Concluding Remarks

Because the application of the traditional deep learning neural network in crop diseases presents the problems of high model complexity, low classification accuracy, inability to be applied to embedded devices, and others, in this study, a recognition model for tomato diseases based on the depthwise separable convolutional neural network was proposed.

From the above experiments and discussions, we observed that the DSCNN model can effectively extract the data features of tomato diseases due to its separation convolution and a series of optimization operations. With the DSCNN model that can reduce the model parameters and calculation amount, the proposed model is well suited for embedded devices in edge computing, dramatically improving the real-time performance of applications. As a future direction, we will deploy this model on edge-embedded device EdgeBoard FZ9A and realize the research and development of a crop diseases and insect pests recognition system aimed at smart agriculture, referencing the model for resource protection and real-time detection, as depicted in Reference [30]. Once implemented, it can be deployed in the smart agriculture greenhouses, and a follow-up control and monitoring application can be developed to facilitate farmers’ timely monitoring of crop growth and operation of diseases and insect pests. For ordinary farmers, they only need to master the use of smartphones, so the learning cost is low.

## Figures and Tables

**Table 1:** Comparison of recognition accuracy and predicted speed.

Model Name	Accuracy (%)	Predicted Speed (s)
LeNet	69.31	1.256
VGGNet	91.75	4.242
DSCNN	89.13	0.239

## Abbreviations

IoT	Internet of Things
CNN	Convolutional neural network
DSCNN	Depthwise separable convolutional neural network
SVM	Support vector machine
